# Unraveling the Global microRNAome Responses to Ionizing Radiation in Human Embryonic Stem Cells

**DOI:** 10.1371/journal.pone.0031028

**Published:** 2012-02-08

**Authors:** Mykyta V. Sokolov, Irina V. Panyutin, Ronald D. Neumann

**Affiliations:** Nuclear Medicine Division, Department of Radiology and Imaging Sciences, Clinical Center, National Institutes of Health, Bethesda, Maryland, United States of America; University of Newcastle upon Tyne, United Kingdom

## Abstract

MicroRNAs (miRNA) comprise a group of short ribonucleic acid molecules implicated in regulation of key biological processes and functions at the post-transcriptional level. Ionizing radiation (IR) causes DNA damage and generally triggers cellular stress response. However, the role of miRNAs in IR-induced response in human embryonic stem cells (hESC) has not been defined yet. Here, by using system biology approaches, we show for the first time, that miRNAome undergoes global alterations in hESC (H1 and H9 lines) after IR. Interrogation of expression levels of 1,090 miRNA species in irradiated hESC showed statistically significant changes in 54 genes following 1 Gy of X-ray exposures; global miRNAome alterations were found to be highly temporally and cell line - dependent in hESC. Time-course studies showed that the 16 hr miRNAome radiation response of hESC is much more robust compared to 2 hr-response signature (only eight genes), and may be involved in regulating the cell cycle. Quantitative real-time PCR performed on some miRNA species confirms the robustness of our miRNA microarray platform. Positive regulation of differentiation-, cell cycle-, ion transport- and endomembrane system-related processes were predicted to be negatively affected by miRNAome changes in irradiated hESC. Our findings reveal a fundamental role of miRNAome in modulating the radiation response, and identify novel molecular targets of radiation in hESC.

## Introduction

MicroRNAs (miRNAs) are considered to constitute a class of small noncoding RNAs vitally involved in regulation of gene expression and signal transduction [1]. Published data suggest miRNAs act as post-transcriptional regulators that may control the expression of about 60% of human genes [2] by means of messenger RNA (mRNA) decay and/or translational repression [3]. Deregulated expression of miRNAs was shown to underlie many diseased states; and miRNAome patterns prove to be highly specific, in many cases accurately reflecting the stage and prognosis of disease [4], [5]. Increasing body of evidence implies changes in miRNA expression profiles after genotoxic stress exposures, including ionizing radiation (IR) [6], [7], [8]. Previous studies analyzed miRNA expression following IR either in fully differentiated human normal somatic cells/artificial 3D tissues [9], [10], [11], [12], [13], [14] or in cancerous cells [15], [16], [17], [18], [19], [20]. Human embryonic stem cells (hESC) represent pluripotent cells with unique capabilities to differentiate into virtually all cell types of a human body. Human stem cells are believed to be endowed with mechanisms to ensure the superior genome fidelity that are just begun to be explored systematically [21], [22]. Exposures to medical, environmental or accidental sources of radiation can challenge the human well-being in the exposed populations, including pregnant women. We, and others, recently studied the IR response of hESC extensively [23], [24], [25], [26], [27], [28], [29]. However, none of the published papers focused on elucidating the miRNA signatures in hESC after IR exposures. Moreover, since the human genome is estimated to encode more than 1,000 of distinct miRNA species, it is imperative to employ high-throughput methodologies to fully analyze the role of miRNA species in biological processes. To the best of our knowledge, only one recent report analyzed miRNAome changes in irradiated human cells at a whole genome-wide level [20].

In the present work, we aimed to study the effects of IR on global miRNAome in hESC, to perform prediction of the biological processes/themes affected by miRNAome changes, and to develop a miRNA-based gene expression signature specific for irradiated, but not for non-irradiated, hESC. For the first time, we found that miRNAome undergoes genome-wide alterations in hESC after IR. All miRNAs published in the Sanger miRBase release version 15.0 (http://microrna.sanger.ac.uk/sequences/index.shtml) were interrogated. The expression levels of 1,090 miRNA species in irradiated hESC showed statistically significant changes in 54 genes following 1 Gy of X-ray exposures (p<0.05). We found that many miRNA species were modulated in a cell line-specific manner in hESC after IR, with H1 cell line being more radiation responsive than H9. Hierarchical clustering, class prediction and Gene Ontology analysis were performed to characterize hESC miRNAome response to radiation in more detail. Positive regulation of differentiation-, cell cycle-, ion transport- and endomembrane system-related processes were predicted as being negatively affected by alterations in miRNAome in irradiated hESC.

## Materials and Methods

### 1. Cell culture and treatments

Human ESCs (H1 and H9 cell lines, WiCell, Madison, WI, passage 35–40; BG01V line, ATCC, Manassas, VA) were routinely cultured in mTeSR-1 medium (Stemcell Technologies, Vancouver, Canada) using cell culture vessels coated with BD Matrigel hESC-qualified Matrix (BD Biosciences, San Jose, CA) at 37°C and 5% CO_2_. Cells were grown following supplier's protocol and as described in [23], [24]. Cell cultures were exposed to 1 Gy of X-ray irradiation using X-RAD 320 Biological Irradiator unit (Precision X-Ray, Inc., North Branford, CT; dose rate about 1 Gy/min; 320 kV, 12.5 mA); then the cells were returned to CO_2_ incubator and harvested at 2 h and 16 h post-irradiation for analysis. The mock-irradiated cells for each time-point were used as a control. For functional analysis of *hsa-mir-575*, both gain- and loss-of-function studies were carried out. For overexpression studies, specific *hsa-mir-575* mirVana miRNA mimic transfection experiments were performed with H1 hESC cultures according to manufacturer's protocol (Applied Biosystems, Carlsbad, CA). MirVana miRNA inhibitor (Applied Biosystems, Carlsbad, CA) was used to knock-down *hsa-mir-575* expression in a separate set of experiments. Transfection studies, in parallel with negative control miRNA inhibitor experiments, were carried out with Stemfect RNA Transfection kit per vendor's protocol (Stemgent, Cambridge, MA). To examine the viability of hESC in colonies upon *hsa-mir-575* overexpression, cells were incubated at 37°C for 1 h with Hoechst 33342 (8 µg/ml; Molecular Probes, Eugene, OR) and propidium iodide (PI, 20 µg/ml; Sigma, St. Louis, MO) as in [25]. Cell colonies were visualized using a fluorescence microscope (Axioplan 2, Zeiss, Thornwood, NY) equipped with a fluorescent light source.

### 2. RNA sample preparation, probe labeling and DNA microarray procedure

The extraction of total RNA was performed with miRNeasy kit (Qiagen, Valencia, CA) per manufacturers' instructions. The amount and quality of RNA samples were assessed on the Agilent 2100 Bioanalyzer with RNA 6000 Nano Reagents and Supplies (Agilent, Santa Clara, CA). Subsequently, 0.5 µg of total RNA was used in each reaction to generate labeled samples with miRCURY LNA microRNA Hy5 Power labeling kit (Exiqon, Woburn, MA). MiRNA spike control was added to the RNA samples prior to the labeling reactions following the manufacturer's protocol. The labeled targets corresponding either to experimental or control samples were separately hybridized to “3D-Gene” oligo microarrays provided by the manufacturer's (Toray Industries Inc., Tokyo, Japan; miRBase release 15.0) containing 1,090-elements spotted in duplicate using Takara Hybridization chambers (Takara Bio, Inc., Japan). Protocols for microarray hybridization and washing were as provided by manufacturer. Hybridized DNA microarrays were scanned on an Axon GenePix DNA microarray scanner (Molecular Devices, Inc., Sunnyvale, CA), and TIFF images were subsequently generated for further analysis. The sample labeling, hybridization, “3D-Gene” array washing and scanning were conducted by Toray Industries Inc. (Tokyo, Japan).

### 3. Microarray data analysis

Analyses were performed using BRB-ArrayTools Version 4.2.0 developed by Dr. Richard Simon and BRB-ArrayTools Development Team (Biometric Research Branch, National Cancer Institute, NIH). Time-matched irradiated versus sham-irradiated samples were used to determine the radiation–responsive miRNA species for each data point. The data from the microarray was collected and analyzed in accordance to the Minimum Information About a Microarray Experiment (MIAME) guidelines. MIAME-compliant raw data for this series of experiments have been deposited in the ArrayExpress database maintained by the European Bioinformatics Institute (accession no. E-MEXP-3366). Differentially expressed miRNA genes were identified using random-variance *t*-test [30] and as in [31], [32]. Changes in gene expression were considered statistically significant if the p values for corresponding genes were less than 0.05.

### 4. Prediction of miRNA targets

Candidate miRNA species with a microarray intensity signal ≥100 and *p*-value≤0.05, identified as being differentially expressed after IR exposures with BRB-ArrayTools, were chosen for target prediction analysis. MiRanda database was used to predict miRNA targets [33]. Targets were input to the Database for Annotation, Visualization and Integrated Discovery (DAVID) [34], [35] themes analysis. Biological themes/processes were determined using the Functional Annotation Clustering feature using *p*-value less than 0.05.

### 5. Quantitative real-time PCR

Total RNA samples used for the microarray analysis were reverse transcribed to cDNA template using RT-specific primers (Applied Biosystems, Carlsbad, CA). Then, TaqMan miRNA assays were performed in triplicate using miRNA Cells-to-C_t_ kit reagents (Applied Biosystems) per manufacturer's instructions. The U6 snRNA was used as an internal control. RT-PCR was performed on iCycler iQ (Bio-Rad, Inc., Hercules, CA) in 20-µl reactions by using TaqMan Assay-on-Demand primers/probe sets (Applied Biosystems) for the following miRNA genes: *hsa-miR-302b*, *hsa-miR-575*, *hsa-miR-1274b*, *hsa-miR-1915* and *hsa-miR-1973*. In a separate set of experiments, H1 hESC cultures transfected either with *hsa-mir-575* mirVana miRNA mimic or miRNA inhibitor were subjected to lysis 5 days post-transfection. TaqMan gene expression studies for *POU5F1* and *SOX2* were carried out in triplicate with Cells-to-C_t_ kit reagents (Applied Biosystems), and 18S RNA expression was used as a reference. Quantitative RT-PCR data were analyzed as in [32].

## Results and Discussion

To identify radiation inducible miRNA species and characterize changes in miRNAome after IR in hESC, we exposed both H1 and H9 cell lines to 1 Gy of X-rays. These cell lines are most extensively studied among hESC. Our miRNA microarray data analysis revealed that 53 and six miRNAs, were differentially expressed in irradiated H1 and H9 cells respectively, compared to corresponding sham-irradiated hESC cultures (Figure S1). These results indicate that miRNA expression profiles of hESC after IR exposures are largely cell line-dependent.

**Figure 1:**
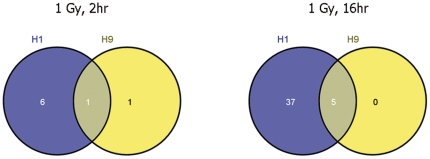
Venn diagrams of significantly upregulated miRNA species in human embryonic stem cells after IR exposures (p<0.05). Comparison of the dynamics of IR response between H1 and H9 cell lines.

Further bioinformatics analyses revealed that only seven miRNA genes were differentially expressed in H1 at the “early” (2 hr) time point post-IR (p<0.05) (Figure 1); among them, only four miRNA species showed more than 1.5 – fold induction compared to sham-irradiated cells (*hsa-miR-15b*, *hsa-miR-1274b*, *hsa-miR-302b* and *hsa-miR-1973*) (Table 1). Gene expression studies showed that IR-induced alterations in miRNAome in H9 cell line (2 hr post-IR) involve the up-regulation of only two miRNA genes, namely, *hsa-miR-1973* and *hsa-miR-92a* (Table 2). In both cell lines, the level of upregulation of miRNA species at this time point was less or equal to 2-fold over corresponding non-irradiated baseline values. No significant down-regulation of miRNA was observed at 2 hr following IR exposures. Interestingly, lack of statistically proven repression of any miRNA species at relatively early 4 hr post 1.25 Gy IR exposure of human blood was reported recently [13].

**Table 1:** Differentially expressed miRNA species in H1 hESC at 2 hr post 1 Gy of X-ray exposures (p<0.05).

Name	p-value	Fold change 2 hr
hsa-miR-15b	0.0136	2.018
hsa-miR-1274b	0.0161	1.789
hsa-miR-302b	0.0107	1.623
hsa-miR-1973	0.0126	1.504
hsa-miR-720	0.0151	1.484
hsa-miR-1274a	0.0385	1.480
hsa-miR-20a	0.0007	1.475

**Table 2:** Differentially expressed miRNA species in H9 hESC at 2 hr post 1 Gy of X-ray exposures (p<0.05).

Name	p-value	Fold change 2 hr
hsa-miR-1973	0.0309	1.732
hsa-miR-92a	0.0193	1.548

On the contrary, at 16 hr timepoint after IR, we observed both statistically significant up-regulation (Table 3) and repression (Table 4) of select miRNA species in H1 hESC. Irradiated H1 cells showed up-regulation of 42 miRNA genes, and down-regulation of only eight miRNAs at this time post 1 Gy IR exposure (p<0.05). A much higher magnitude of changes in the level of expression of IR-modulated miRNAs was observed as part of the “late” hESC response to IR (Table 3); 33 miRNA species were induced more than 2 – fold in irradiated H1 compared to control sham-exposed cell cultures. In contrast, we found that only five miRNAs were significantly overexpressed in H9 cell line, with a single miRNA species, *hsa-miR-575*, being overexpressed more than 2 fold (p<0.05) (Table 5).

**Table 3:** Up-regulated miRNA species in H1 hESC at 16 hr post 1 Gy of IR exposures (p<0.05).

Name	p-value	Fold change 16 hr
hsa-miR-575	0	3.817
hsa-miR-1915	0.0003	3.322
hsa-miR-3195	0.0004	3.105
hsa-miR-3196	0.0002	2.845
hsa-miR-744	0.0018	2.843
hsa-miR-1908	0.0002	2.804
hsa-miR-1975	0.0069	2.780
hsa-miR-663	0.0005	2.762
hsa-miR-3178	0	2.744
hsa-miR-149*	0.002	2.728
hsa-miR-4257	0.0002	2.726
hsa-miR-762	0.0005	2.671
hsa-miR-1469	0.0003	2.668
hsa-miR-4281	0	2.663
hsa-miR-614	0.0003	2.646
hsa-miR-4327	0.0004	2.609
hsa-miR-2861	0.0022	2.581
hsa-miR-1268	0.0014	2.555
hsa-miR-24	0.0013	2.492
hsa-miR-1228*	0.0002	2.461
hsa-miR-638	0.0012	2.401
hsa-miR-1275	0.0034	2.396
hsa-miR-711	0.0001	2.385
hsa-miR-671-5p	0.0005	2.340
hsa-miR-3197	0.0004	2.312
hsa-miR-1909	0	2.205
hsa-miR-3141	0.0001	2.167
hsa-miR-3180-3p	0	2.130
hsa-miR-1308	0.0009	2.112
hsa-miR-92b*	0.0005	2.105
hsa-miR-187*	0.0009	2.055
hsa-miR-513a-5p	0.0081	2.032
hsa-miR-1973	0.0126	2.028
hsa-miR-370	0.0015	1.943
hsa-miR-494	0.0063	1.866
hsa-miR-1914*	0.0004	1.758
hsa-miR-940	0.0189	1.725
hsa-miR-1274b	0.0161	1.631
hsa-miR-1260b	0.0007	1.547
hsa-miR-675	0.0311	1.497
hsa-miR-874	0.0159	1.418
hsa-miR-612	0.0219	1.404

**Table 4:** Down-regulated miRNA species in H1 hESC at 16 hr post 1 Gy of IR exposures (p<0.05).

Name	p-value	Fold change 16 hr
hsa-miR-886-3p	0.0448	0.712
hsa-miR-93	0.0013	0.706
hsa-miR-302d	0.003	0.681
hsa-miR-17	0.028	0.639
hsa-miR-20a	0.0007	0.626
hsa-miR-302a	0.0004	0.586
hsa-miR-20b	0.0008	0.567
hsa-miR-302b	0.0107	0.449

**Table 5:** Differentially expressed miRNA species in H9 hESC at 16 hr post 1 Gy of X-ray exposures (p<0.05).

Name	p-value	Fold change 16 hr
hsa-miR-575	0.0227	2.171
hsa-miR-513a-5p	0.0383	1.796
hsa-miR-711	0.0283	1.686
hsa-miR-1973	0.0309	1.535
hsa-miR-1275	0.0387	1.501

**Figure 2:**
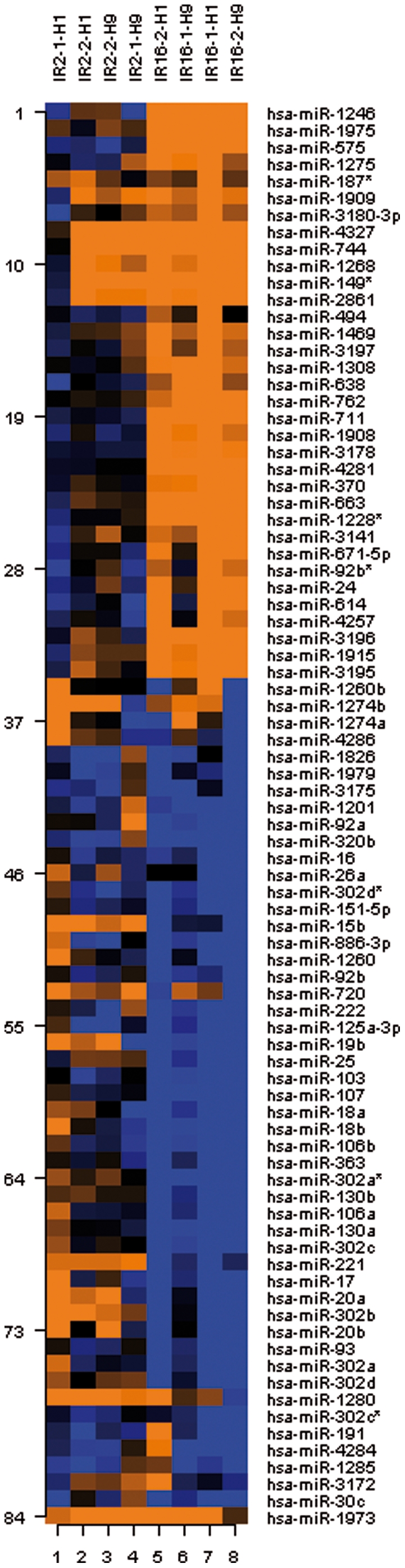
Heatmap representation of differentially expressed miRNA species in human embryonic stem cells after IR exposures (p<0.05). Clustering analysis was performed on all IR-modulated miRNAs with the signal intensity more than 50.

To identify the factors that affect the patterns of microRNAome changes after IR exposures, all experimental samples were subjected to hierarchical clustering (Figure 2). This analysis grouped hESC samples in accordance to IR exposure conditions, with the most dramatic changes in miRNAome occurring as a result of dynamic of cellular response to radiation. The timing of post-IR exposure response, and not a cell type – specific changes, was the most prominent determinant of the clustering of samples analyzed.

Our recent study demonstrated no statistically significant downregulation of mRNA gene transcripts after 1 Gy of IR exposure in H9 cells [24]. Intriguingly, in the present study we observed a few miRNAs that were upregulated in H9 under these same conditions (Table 2 and Table 5). Since miRNAs are considered to be one of the powerful epigenetic means to silence gene expression, it may seem puzzling at first glance. However, it is thought that in mammals the primary mode of miRNA-mediated gene repression is through inhibition of translation, not direct mRNA target degradation [36]. Therefore, it could explain the lack of downregulation of any protein-coding transcripts observed in our previous work [24]. The repression of genes involved in hESC differentiation after IR exposures may explain the maintenance of pluripotentiality in surviving irradiated hESC that we reported before [25]. Changes in cell cycle profile of IR-exposed hESC [24] may also be the consequence of miRNAome alterations. For example, one of the key genes responsible for cell cycle progression and about 26% of all phosphorylation events in hESC is CDK1/2 [37], which we found to be the major predicted target of the most highly upregulated miRNA (*hsa-miR-575*) in our present study. Interestingly, *hsa-miR-575* is known to be overexpressed in some aggressive human cancers [38], and is downregulated upon human leukemic HL-60 cell differentiation induced by 4-hydroxynonenal, a product of lipid peroxidation [39]. We sought to investigate the role of *hsa-miR-575* in hESC in more detail. Our studies aimed to mimic the overexpression of this miRNA species showed no evidence for an increase in cell killing in H1 hESC cultures (Figure S2). Moreover, we did not observe signs of differentiation in these cultures since continuous expression of markers of pluripotency, such as *POU5F1* and *SOX2*, was evident at least 5 days post-transfection (Table S1). Interestingly, upon treatment of irradiated H1 cells with *hsa-miR-575* inhibitor as part of our loss-of-function studies, we observed substantial down-regulation of *POU5F1* and upregulation of *SOX2* (Table S1). Both genes are known to play an important role in maintenance of pluripotency in undifferentiated hESC, and major alterations in their expression levels may result in differentiation [40], [41]. Our results may support the assumption that upregulation of *hsa-miR-575* in irradiated hESC serves to prevent differentiation of these cells, but additional studies into the exact function of *hsa-miR-575* are needed to be performed in a future.

In general, the majority of differentially expressed miRNAs that we identified in our global screen of irradiated hESC are not well-characterized yet. However, the involvement of some IR-responsive miRNAs in biological processes was already reported. For example, the most highly induced miRNA in H1 cells at 2 hr post-1 Gy of IR exposures, *hsa-miR-15b*, was shown to sensitize human cancer cells to apoptosis [42]. This might be relevant for cell fate choice in hESC as well, since massive apoptosis was observed in irradiated hESC after 1 Gy of X-ray exposures [25]. In addition, the up-regulation of this miRNA species could influence many other aspects of cellular homeostasis: *miR-15b* is overexpressed in bronchioalveolar lung stem cells [43], is a direct transcriptional target of E2F1 modulating cell cycle progression [44], and is known to affect cell metabolism through decreasing ATP levels [45].

*Hsa-mir-302b* was shown to be highly expressed in hESC [46] and is directly responsible for “stemness” characteristics in human cells being part of stem cell-enriched mir-302-367 cluster [47], [48]. Mir-302 species were determined to be highly integrated with Oct4/Sox2 transcriptional machinery in hESC being partly responsible for unique abbreviated cell cycle profile in hESCs [49]. The modest upregulation of this miRNA species following IR exposures may prevent hESC from undergoing spontaneous differentiation. Other IR-modulated miRNA species, such as *hsa-mir-20a*, is a component of yet another stem cell-enriched family, namely, mir-17-92, which is implicated in modulating E2F activity on cell cycle progression [50] and in the repression of execution of cellular senescence program [51], [52]. Hsa-mir-149* also regulates E2F1 activity and exerts its function through induction of apoptosis [53]. One more hESC highly expressed miRNA, *hsa-mir-1909*, was implicated in hESC physiology by targeting Notch [54].

A large number of overexpressed IR-responsive miRNAs that we identified in our work were found to be deregulated in human cancers, such as *hsa-mir-513* [55], *hsa-mir-744* [56], *hsa-mir-92a* [57], [58], hsa-mir-1228* [59], *hsa-mir-671-5p* [60], *hsa-mir-638* [38], *hsa-mir-370* [61], and *hsa-mir-675* [62]. *Hsa-mir-663* is involved in inflammatory conditions and suppression of cell proliferation [63], [64] which can underlie hESC response to IR exposures. Interestingly, two of significantly upregulated miRNAs after IR exposures in hESC, such as *hsa-mir-1275* and *hsa-mir-494*, were recently found to localize to mitochondria [65]. Given the importance of mitochondrial compartment in the maintenance of stem cell homeostasis and responses to different stress factors [66], the functional validation of targets of these miRNA species might prove to be instrumental in unraveling the underpinnings of hESC unique biological capabilities.

We observed several miRNA species that were downregulated following irradiation (Table 4). Repression of *hsa-mir-93* could block cell differentiation after IR exposures in part by relieving STAT3 [67]. Other biological effect of decreased expression of *hsa-mir-93* at 16 hr post-IR in H1 could be a decreased cell survival [68], probably as a result of modulation of its targets such as E2F1 and *CDKN1A* [69]. Interestingly, *CDKN1A* was found to be a target for other down-regulated miRNA species we identified in our present study, namely, *hsa-mir-302a*, *hsa-mir-302b*, and *hsa-mir-302d* [70]. With *CDKN1A* being one of the key radiation-responsive genes in human cells, the possible involvement of miRNAome alterations in its regulation after IR definitely merits further investigations.

We attempted to compare our data on global miRNAome alterations in irradiated hESC with published datasets. However, since the knowledge about changes in miRNA levels in human cells following irradiation is very limited to date, we were able to identify only a few miRNA species in our subset of IR-modulated miRNAs which were reported before as responsive to IR exposures. Among them, we found *hsa-mir-20a*, shown to be IR-responsive in fully differentiated human endothelial cells [10] and implicated in regulation of IR-induced premature senescence [52]; *hsa-mir-24*, a member of the miR-23b cluster that interferes with TGF-β expression and shown to be up-regulated after IR [20]. Mir-24 is implicated in halting cell cycle progression and inhibition of apoptosis [71], and impairment of DDR and senescence program execution [72], [73]. In addition, *hsa-mir-744* and *hsa-mir-17* were also found to be deregulated by IR exposures by others [20]. Our studies with BG01V line of hESC, considered being karyotypically abnormal, identified *hsa-mir-1915* and *hsa-mir-1274b* as differentially expressed at 2 hr and 16 hr post-IR, respectively (Table S2). We believe that further experiments will examine the relative contribution of cell type specificity to a repertoire of IR-modulated miRNAs at a whole-genome level in various human cells.

We analyzed the possible targets of differentially expressed miRNAs by running MiRanda algorithm search. The results are presented in Tables S3, S4, S5, S6, S7. At least some of the highly scored targets may represent an integrated network with miRNAome and act in concert in irradiated hESCs; for example, LIN9, a putative target of *hsa-mir-1973*, regulates MYB which is by itself is predicted to be a target of *hsa-mir-15b* (Table S3), and crucially affects the cell cycle machinery through the cyclins and CDK1 [74]. Hence, modulation of regulators of key biological processes by alterations in miRNAome may represent a powerful strategy used by IR-exposed hESC to cope with genotoxic stress.

Despite numerous recent advances, the human miRNAome still remains mainly unexplored regarding the physiological function of specific miRNA species within cells. It should be noted that deciphering the function of individual miRNAs is challenging. There are many families comprising microRNAs differing only in one-two nucleotides which make a functional assignment to these redundant genes a complicated task. Each miRNA is thought to target numerous putative transcripts that have non-related functions, thereby the experimental validation of specific targets for miRNAs is still in its infancy. Therefore, determining which biological processes might be prime candidates for miRNA-mediated regulation of gene expression after IR exposures might prove to be more informative than querying individual miRNA-mRNA pairs. To this end, we performed Gene Ontology (GO) analysis of biological themes/processes. The aim of the GO analysis was to predict the effect of overexpressed miRNAs on cellular functions. The downregulated genes predicted to be targeted by the radiation-induced miRNAs, were uploaded to the DAVID GO database, and gene-set enrichment analysis was carried out in the biologic process/molecular function/cellular component categories. The results of the GO analysis of the biological themes overrepresented in the pools of the genes predicted to be targeted by miRNAs are shown in Table 6 and Table 7. Our data indicate that IR exposures of H1 and H9 hESCs could result in an increased miRNA control of genes involved in positive regulation of cell differentiation, cell projection, transcription activation, alternative splicing, cell death and cell cycle regulation.

**Table 6:** Gene Ontology analysis of affected biological themes based on predicted miRNA targets in H1 cell line.

Exposures	Overrepresented categories	EASE score
1 Gy, 2 hrs	Positive regulation of cell differentiation	2.0E-4
	Cell projection	6.3E-4
	Ion binding	0.004
	Transcription activator activity	0.0041
	Alternative splicing	0.0067
	Cell cycle	0.01
	Response to endogenous stimulus	0.013
	Pore complex	0.015
	Response to hormone stimulus	0.019
	Endomembrane system	0.026
	Regulation of transcription	0.045
1 Gy, 16 hrs	Protocadherin gamma	5.0E-19
	Alternative splicing	1.6E-16
	Membrane-enclosed lumen	2.3E-5
	Regulation of mRNA stability	2.9E-5
	Cell projection	6.2E-5
	Positive regulation of cell differentiation	2.2E-4
	Positive regulation of transcription from RNAP2 promoter	5.1E-4
	mRNA stabilization	7.6E-4
	Muscle tissue development	0.0027
	Positive regulation of cell proliferation	0.0032
	Heart development	0.0091

**Table 7:** Gene Ontology analysis of affected biological themes based on predicted miRNA targets in H9 cell line.

Exposures	Overrepresented categories	EASE score
1 Gy, 2 hrs	Ion transport	0.0027
	Ion binding	0.0071
	Membrane-bounded vesicle	0.012
	Heart development	0.018
	Cell projection part	0.019
	Endomembrane system	0.02
	Positive regulation of cell differentiation	0.022
	Cytoskeleton	0.035
	Cell death	0.049
1 Gy, 16 hrs	Mesenchyme development	4.6E-4
	Cell cycle control	5.2E-4
	Negative regulation of nucleic acid metabolic process	6.9E-4
	Negative regulation of macromolecule biosynthesis	0.0014
	Negative regulation of transcription	0.0019
	Heart development	0.0056
	Cell morphogenesis involved in differentiation	0.011
	Cytoskeleton	0.048

To partially validate miRNAome changes we obtained in our study, we compared data on miRNAs expression with two independent techniques, that is, miRNA microarray platform and quantitative RT-PCR (Table 8). In general, we observed a good concordance between these datasets, thus confirming the robustness of our approach.

**Table 8:** Verification of miRNA microarray data with Taqman qRT-PCR. Shown are means and standard errors for corresponding values obtained by two techniques.

	Hsa-miR-302b		Hsa-miR-575		Hsa-miR-1274b		Hsa-miR-1973	
	Array	qRT-PCR	Array	qRT-PCR	Array	qRT-PCR	Array	qRT-PCR
H1 1 Gy 2 hrs	1.66±0.05	1.32±0.25	1.10±0.06	1.12±0.47	1.83±0.08	0.90±0.44	1.54±0.27	0.77±0.17
H1 1 Gy 16 hrs	0.48±0.09	0.35±0.05	3.96±0.33	2.90±2.43	1.80±0.41	2.18±0.88	2.21±0.97	1.75±0.46
H9 1 Gy 2 hrs	1.40±0.28	1.62±0.77	1.05±0.15	1.05±0.35	1.38±0.41	1.52±0.15	1.85±0.76	1.31±0.38
H9 1 Gy 16 hrs	1.07±0.02	1.17±0.36	2.26±0.33	2.82±0.79	1.17±0.12	0.90±0.73	1.55±0.06	2.59±0.60

In summary, we found that microRNAome of human embryonic stem cells undergoes global alterations following ionizing radiation exposures. We showed that the gene expression signature for global miRNAome alterations at a “late”, 16 hr time point is significantly different in comparison to that of “early” response. The changes that we observed in the levels of miRNA species constituting the whole human microRNAome are substantially cell line-dependent, with H1 cell line of hESC being more responsive to IR than H9 line. Surprisingly, there were just a few H9-specific IR-modulated miRNA species compared to H1. Since these two cell lines are most thoroughly analyzed to date among all published hESC lines, it might be of interest to probe miRNAome of these lines under more various experimental settings in a future. We observed consistent patterns of miRNA response to IR exposures, including several miRNA species that were systematically differentially expressed at both time intervals. But the majority of IR-modulated miRNA showed a time-dependent response in both H1 and H9 hESCs, with a more robust response occurring at a late time point. It should be noted that the degree of target repression imposed by miRNAs is probably quantitatively modest since most of specific miRNA species endogenous targets is usually downregulated by less than 50% [75]. Given these considerations, most proteins comprising the human proteome presumably remain effective over this degree of inhibition. Therefore, future studies will focus on how alterations in miRNAome affect the proteome of hESC following IR-exposures, and how it translates to the ultimate cell fate of these unique human cells that show great promise in both disease modeling and cell regenerative therapy approaches.

The strength of our study is based upon the comprehensive coverage of the whole human miRNAome in an experimental platform that we employed, in profiling the changes in miRNAs in two different hESC lines, and in analyzing the dynamics of response of hESC to IR exposures at the level of miRNAome. We identified a number of radiation-responsive miRNA species with as yet unknown functions that provide a broad avenue to future research in this area. We attempted to outline some of hypothesis that can foster the investigation of functional relevance of miRNAome alterations in hESC. Our study reveals new insights into how hESC respond to genotoxic stress, in particular, to IR exposures resulting in global alterations in microRNAome in these cells. The findings may contribute to improved understanding of the biology of hESC, and the mechanisms underlying enhanced genomic maintenance in hESC.

## Supporting Information

Figure S1 Venn diagram of a total number of differentially expressed miRNA species in human embryonic stem cells after IR exposures (p<0.05).
(TIF)

Figure S2 H1 hESC culture staining for viability upon *hsa-mir-575* changes in gene expression studies. Cell cultures were stained with Hoechst 33342 (shown in blue) and propidium iodide (in red). A – control, 0 Gy; B – mock transfection; C – *hsa-mir-575* mimic transfection; D – 1 Gy, 24 hrs post- IR.
(TIF)

Table S1 Taqman qRT-PCR on cultured H1 hESCs. Shown are means and standard errors for gene expression changes obtained following *hsa-mir-575* expression modulation in comparison to mock-treated cell cultures.
(DOC)

Table S2 Taqman qRT-PCR on cultured BG01V hESCs. Shown are means and standard errors for miRNA expression changes obtained for indicated timepoints after irradiation in comparison to mock-treated cell cultures.
(DOC)

Table S3 Up-regulated (>1.5 - fold) miRNA genes (1 Gy, 2 hr) in H1 as determined by microarray analysis (p<0.05).
(DOC)

Table S4 Selection of top 10 up-regulated miRNA genes (1 Gy, 16 hr) in H1 as determined by microarray analysis (p<0.05).
(DOC)

Table S5 Down-regulated (>1.5 - fold) miRNA genes (1 Gy, 16 hr) in H1 (p<0.05).
(DOC)

Table S6 Up-regulated (>1.5 - fold) miRNA genes (1 Gy, 2 hr) in H9 (p<0.05).
(DOC)

Table S7 List of up-regulated (>1.5 - fold) miRNA genes (1 Gy, 16 hr) in H9 (p<0.05).
(DOC)
