# Supplementary figures and images for: Unraveling the Global microRNAome Responses to Ionizing Radiation in Human Embryonic Stem Cells
Source: PLoS One. 2012 Feb 8;7(2):e31028. doi: 10.1371/journal.pone.0031028 (PMC3275573; doi:10.1371/journal.pone.0031028)

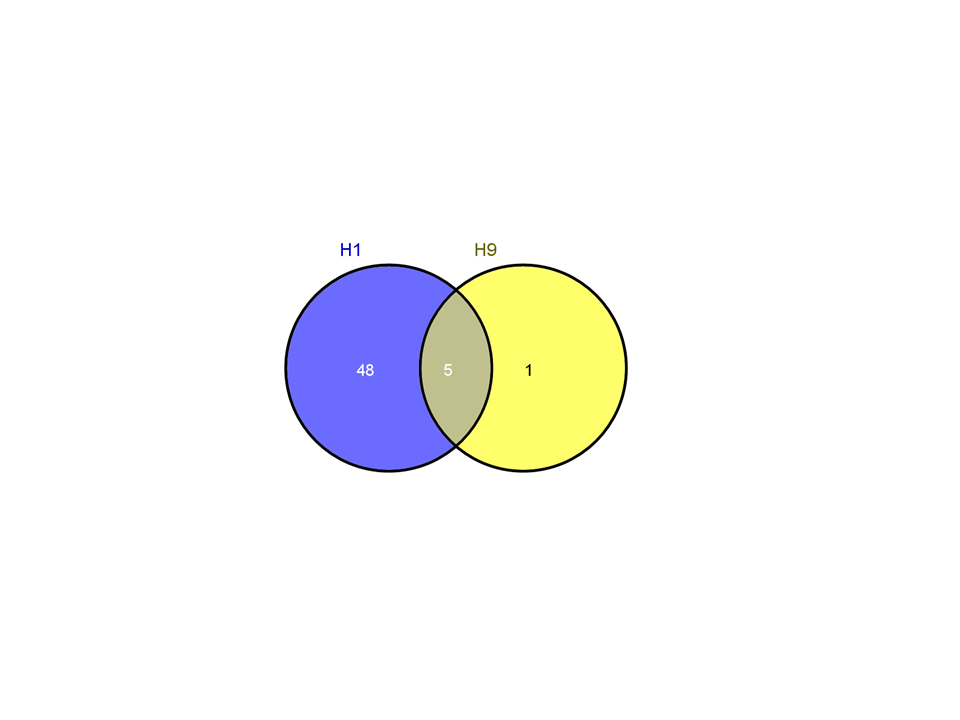

Supplement: Figure S1 — Venn diagram of a total number of differentially expressed miRNA species in human embryonic stem cells after IR exposures (p<0.05). (TIF) [file pone.0031028.s001.tif]

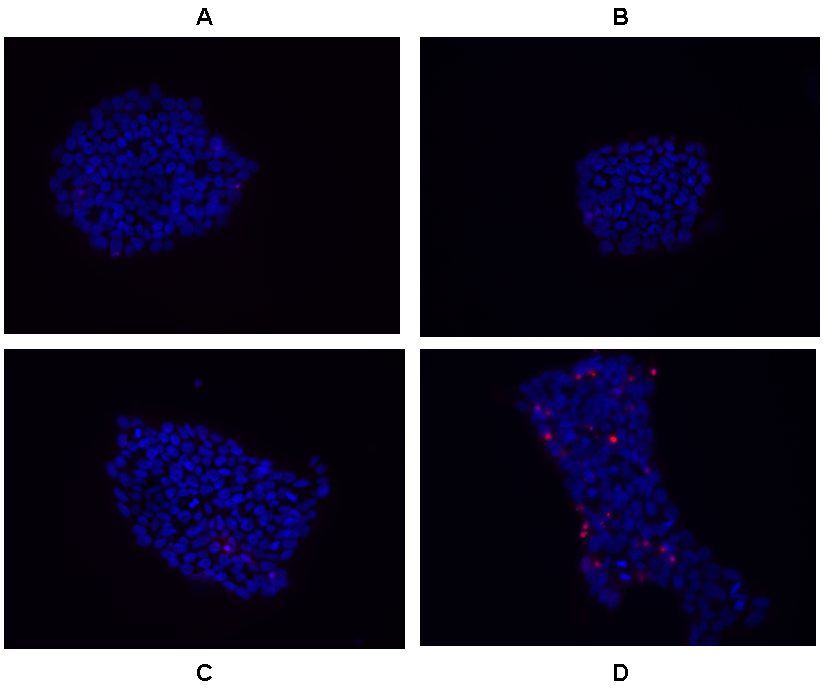

Supplement: Figure S2 — H1 hESC culture staining for viability upon hsa-mir-575 changes in gene expression studies. Cell cultures were stained with Hoechst 33342 (shown in blue) and propidium iodide (in red). A – control, 0 Gy; B – mock transfection; C – hsa-mir-575 mimic transfection; D – 1 Gy, 24 hrs post- IR. (TIF) [file pone.0031028.s002.tif]
