# Supplementary material for: Unraveling the Global microRNAome Responses to Ionizing Radiation in Human Embryonic Stem Cells
Source: PLoS One. 2012 Feb 8;7(2):e31028. doi: 10.1371/journal.pone.0031028 (PMC3275573; doi:10.1371/journal.pone.0031028)
Supplement: Table S1 — Taqman qRT-PCR on cultured H1 hESCs. Shown are means and standard errors for gene expression changes obtained following hsa-mir-575 expression modulation in comparison to mock-treated cell cultures. (DOC) [file pone.0031028.s003.doc]

|  | ***POU5F1*** | ***SOX2*** |
| --- | --- | --- |
| MiRvana miRNA mimic, 5 days post transfection | 1.44 ± 0.77 | 1.08 ± 0.50 |
| MiRvana miRNA inhibitor, 1 Gy, 5 days post transfection | 0.37 ± 0.07 | 19.76 ± 8.19 |
