# Supplementary material for: Unraveling the Global microRNAome Responses to Ionizing Radiation in Human Embryonic Stem Cells
Source: PLoS One. 2012 Feb 8;7(2):e31028. doi: 10.1371/journal.pone.0031028 (PMC3275573; doi:10.1371/journal.pone.0031028)
Supplement: Table S2 — Taqman qRT-PCR on cultured BG01V hESCs. Shown are means and standard errors for miRNA expression changes obtained for indicated timepoints after irradiation in comparison to mock-treated cell cultures. (DOC) [file pone.0031028.s004.doc]

|  | Hsa-miR-302b | Hsa-miR-575 | Hsa-miR-1274b | Hsa-miR-1915 |
| --- | --- | --- | --- | --- |
| 1 Gy 2 hrs | 1.06 ± 0.28 | 1.29 ± 0.20 | 0.94 ± 0.35 | 1.58 ± 0.36 |
| 1 Gy 16 hrs | 1.04 ± 0.39 | 1.14 ± 0.26 | 1.66 ± 0.72 | 1.18 ± 0.14 |
