# Supplementary material for: Unraveling the Global microRNAome Responses to Ionizing Radiation in Human Embryonic Stem Cells
Source: PLoS One. 2012 Feb 8;7(2):e31028. doi: 10.1371/journal.pone.0031028 (PMC3275573; doi:10.1371/journal.pone.0031028)
Supplement: Table S3 — Up-regulated (>1.5 - fold) miRNA genes (1 Gy, 2 hr) in H1 as determined by microarray analysis (p<0.05). (DOC) [file pone.0031028.s005.doc]

| Gene name | Selection of predicted mRNA targets |
| --- | --- |
| *miR-15b* | *UNC80, USP15, CD80, ARIH1, MYB, HMGA2, WEE1* |
| *miR-1274b* | *BAZ2B, SMAP1, SOX21, GATA6* |
| *miR-302b* | *CROT, TGFBR2, OHR, UBE2B, POLK, PLEKHA3* |
| *miR-1973* | *UTRN, AR, NDRG1, SHC4, LIN9, BARD1, CDK2AP1* |
