# Supplementary material for: Unraveling the Global microRNAome Responses to Ionizing Radiation in Human Embryonic Stem Cells
Source: PLoS One. 2012 Feb 8;7(2):e31028. doi: 10.1371/journal.pone.0031028 (PMC3275573; doi:10.1371/journal.pone.0031028)
Supplement: Table S4 — Selection of top 10 up-regulated miRNA genes (1 Gy, 16 hr) in H1 as determined by microarray analysis (p<0.05). (DOC) [file pone.0031028.s006.doc]

| Gene name | Selection of predicted mRNA targets |
| --- | --- |
| *miR-575* | *CDK1, ABCD3, RNF41, WEE1* |
| *miR-1915* | *PBX2, ZNF474, DEAF1, CBARA1* |
| *miR-3195* | *ZNF410, UBE2A, NKX2-5, ALDH3B2* |
| *miR-3196* | *BBC3, ATP1A3, JUND, NKX2-2, LGI3* |
| *miR-744* | *HNRNPC, EIF4A2, CSNK2A1, CDK18, CD44, CD37* |
| *miR-1908* | *ABO, PRRT1, CDK17, NKX2-1, HMGA2* |
| *miR-1975* | *MYO3B, VEGFA, UBE3B, FOXG1* |
| *miR-663* | *ABO, PRRT1, CSNK2A1, ARAF, HMGA2* |
| *miR-3178* | *MYOD1, STAU2, CDK18, EIF4B, CDC6* |
| *miR-149** | *CSNK2A1, EIF3H, UBE2Q1, RALB* |
