# Supplementary material for: Unraveling the Global microRNAome Responses to Ionizing Radiation in Human Embryonic Stem Cells
Source: PLoS One. 2012 Feb 8;7(2):e31028. doi: 10.1371/journal.pone.0031028 (PMC3275573; doi:10.1371/journal.pone.0031028)
Supplement: Table S5 — Down-regulated (>1.5 - fold) miRNA genes (1 Gy, 16 hr) in H1 (p<0.05). (DOC) [file pone.0031028.s007.doc]

| Gene name | Selection of predicted mRNA targets |
| --- | --- |
| *hsa-miR-17* | *STAT3, DYNC1L2, PLEKHA3, SUV420H1, ENPP5, EPHA4* |
| *hsa-miR-20a* | *STAT3, PLEKHA3, SUV420H1, ENPP5, EPHA4* |
| *hsa-miR-302a* | *TGFBR2, CROT, NR4A2, UBE2B, POLK, PLEKHA3, ENPP5* |
| *hsa-miR-20b* | *STAT3, PLEKHA3, SUV420H1, ENPP5, EPHA4* |
| *hsa-miR-302b* | *TGFBR2, CROT, NR4A2, UBE2B, POLK, PLEKHA3* |
