# Supplementary material for: Unraveling the Global microRNAome Responses to Ionizing Radiation in Human Embryonic Stem Cells
Source: PLoS One. 2012 Feb 8;7(2):e31028. doi: 10.1371/journal.pone.0031028 (PMC3275573; doi:10.1371/journal.pone.0031028)
Supplement: Table S7 — List of up-regulated (>1.5 - fold) miRNA genes (1 Gy, 16 hr) in H9 (p<0.05). (DOC) [file pone.0031028.s009.doc]

| Gene name | Selection of predicted mRNA targets |
| --- | --- |
| *hsa-miR-575* | *CDK1, ABCD3, RNF41, WEE1* |
| *hsa-miR-513a-5p* | *ABCB5, GYPA, EPS8, FBXO34, NODAL* |
| *hsa-miR-711* | *GHRHR, STIM1, GATA2, CDK4, EIF2B1* |
| *hsa-miR-1973* | *UTRN, AR, NDRG1, SHC4, LIN9, BARD1, CDK2AP1* |
| *hsa-miR-1275* | *SIRT2, TGFBR3, ACTB, TOP1P1, HNRNPAB* |
